# Two New Local Anæsthetics

**Published:** 1885-10

**Authors:** 


### TWO NEW LOCAL ANÆSTHETICS.

DR. T. J. MAYS, of Philadelphia, in the *Therapeutic Gazette*, gives an account of experiments which demonstrate that the alkaloid brucia has distinct local anæsthetic properties. It is only pure brucia, however, that gives these results—the commercial brucia being little more, he says, than impure strychnia. In the *N. Y. Medical Journal* there is a note on menthol as a local anæsthetic which says, on the authority of Rosenberg, that a twenty to thirty per cent solution of menthol, which is much cheaper than cocaine, is a useful substitute for the latter as an application to mucous surfaces like the nose, pharynx and larynx. The effect is more evanescent than that of cocaine, and appears to be cumulative, somewhat.

In addition to the above, experiments have been made recently with apomorphia, which show that a few drops of a ten per cent solution dropped into a rabbit's eye has identically the same effect cocaine has; and the effect lasts about the same length of time. Chemists say the reason why cafein, the analogue of cocaine, is not equally as serviceable as an anæsthetic, is because it is less soluble. Why seek a substitute for cocaine now that the supply is abundant, and the price has fallen, we believe, to about one-tenth the former price. Cocaine seems to be *the* desideratum long sought.

